# The Administration of Chitosan-Tripolyphosphate-DNA Nanoparticles to Express Exogenous SREBP1a Enhances Conversion of Dietary Carbohydrates into Lipids in the Liver of *Sparus aurata*

**DOI:** 10.3390/biom9080297

**Published:** 2019-07-24

**Authors:** Jonás I. Silva-Marrero, Juliana Villasante, Ania Rashidpour, Mariana Palma, Anna Fàbregas, María Pilar Almajano, Ivan Viegas, John G. Jones, Montserrat Miñarro, Josep R. Ticó, Isabel V. Baanante, Isidoro Metón

**Affiliations:** 1 Secció de Bioquímica i Biologia Molecular, Departament de Bioquímica i Fisiologia, Facultat de Farmàcia i Ciències de l’Alimentació, Universitat de Barcelona, Joan XXIII 27–31, 08028 Barcelona, Spain; 2 Departament d’Enginyeria Química, Universitat Politècnica de Catalunya, Diagonal 647, 08028 Barcelona, Spain; 3 Center for Functional Ecology (CFE), Department Life Sciences, University of Coimbra, Calçada Martins de Freitas, 3000-456 Coimbra, Portugal; 4 Departament de Farmàcia i Tecnologia Farmacèutica, i Fisicoquímica, Facultat de Farmàcia i Ciències de l’Alimentació, Universitat de Barcelona, Joan XXIII 27-31, 08028 Barcelona, Spain; 5 Center for Neuroscience and Cell Biology (CNC), University of Coimbra, Largo Marquês de Pombal, 3004-517 Coimbra, Portugal

**Keywords:** SREBP1, chitosan, nanoparticles, gene delivery, metabolism, *Sparus aurata*

## Abstract

In addition to being essential for the transcription of genes involved in cellular lipogenesis, increasing evidence associates sterol regulatory element binding proteins (SREBPs) with the transcriptional control of carbohydrate metabolism. The aim of this study was to assess the effect of overexpression SREBP1a, a potent activator of all SREBP-responsive genes, on the intermediary metabolism of *Sparus aurata*, a glucose-intolerant carnivorous fish. Administration of chitosan-tripolyphosphate nanoparticles complexed with a plasmid driving expression of the N-terminal transactivation domain of SREBP1a significantly increased SREBP1a mRNA and protein in the liver of *S. aurata*. Overexpression of SREBP1a enhanced the hepatic expression of key genes in glycolysis-gluconeogenesis (glucokinase and 6-phosphofructo-2-kinase/fructose-2,6-bisphosphatase), fatty acid synthesis (acetyl-CoA carboxylase 1 and acetyl-CoA carboxylase 2), elongation (elongation of very long chain fatty acids protein 5) and desaturation (fatty acid desaturase 2) as well as reduced nicotinamide adenine dinucleotide phosphate production (glucose-6-phosphate 1-dehydrogenase) and cholesterol synthesis (3-hydroxy-3-methylglutaryl-coenzyme A reductase), leading to increased blood triglycerides and cholesterol levels. Beyond reporting the first study addressing in vivo effects of exogenous SREBP1a in a glucose-intolerant model, our findings support that SREBP1a overexpression caused multigenic effects that favoured hepatic glycolysis and lipogenesis and thus enabled protein sparing by improving dietary carbohydrate conversion into fatty acids and cholesterol.

## 1. Introduction

The sterol regulatory element binding protein (SREBP) family of transcription factors has a major role in regulating the expression of genes that control cellular lipid biosynthesis [1,2]. SREBPs are synthesized as membrane-bound precursors that remain anchored to the endoplasmic reticulum membrane. The C-terminal domain of SREBP precursors associates with a sterol sensor, the SREBP-cleavage activating protein (Scap), which in turn interacts with insulin-induced gene (Insig) protein [3,4]. Sterol deprivation drives the SREBP/Scap complex to the Golgi apparatus, where a two-step cleavage process that involves site-1 and site-2 proteases (S1P and S2P, respectively) releases the N-terminal domain of SREBPs, which are transcriptionally active basic-helix-loop-helix leucine zipper (bHLH-Zip) factors [5]. After dimerization, mature SREBPs translocate to the nucleus and transactivate target genes by binding to sterol regulatory element (SRE) sequences [6].

The activity of alternate promoters in the *srebf1* gene generates two isoforms of SREBP1 in mammals (SREBP1a and SREBP1c), while a second gene, *srebf2*, encodes for the SREBP2 protein [7]. Alternatively spliced variants have been reported for both genes [8,9,10]. SREBP1c primarily transactivates genes required for fatty acid and triglyceride synthesis, while SREBP2 regulates the transcription of genes associated with cholesterol metabolism [7,11]. SREBP1a exhibits stronger transcriptional activity than SREBP1c due to the presence of a longer N-terminal transactivation domain, and it is a potent activator of all SREBP-responsive genes [12,13,14]. Recent advances highlight the role of insulin-dependent activation of SREBPs through the PI3K-Akt-mTOR pathway in pathophysiological processes associated with impaired lipid metabolism, such as diabetes mellitus, non-alcoholic fatty liver disease, neurogenerative diseases, innate immunity and cancer [1,7].

In addition to the well-known effect of SREBP1 isoforms on lipid metabolism [1,2,7], evidence supports the involvement of SREBP1 in glucose metabolism. In the mammalian liver, SREBP1c mediates insulin-dependent upregulation of hexokinase II and glucokinase (GK) transcription by transactivating the promoters of both genes [15,16,17,18]. Furthermore, overexpression of SREBP1a in transgenic mice decreases the hepatic gluconeogenic flux due to the suppression of phosphoenolpyruvate carboxykinase and glucose-6-phosphatase (G6Pase) transcriptional activation mediated by hepatocyte nuclear factor-4α [19]. In a carnivorous fish, gilthead sea bream (*Sparus aurata*), we previously reported that SREBP1 upregulates the transcription of GK and 6-phosphofructo-2-kinase/fructose-2,6-bisphosphatase (PFKFB1) by binding to SRE boxes located in their respective gene promoters [20,21].

Carnivorous fish make efficient use of dietary protein to provide amino acids that serve as building blocks for the synthesis of new proteins and catabolic substrates to produce energy. On the contrary, glucose resulting from dietary carbohydrate digestion and free sugars are metabolised markedly slower than in mammals, and give rise to prolonged hyperglycemia [22]. Dysregulation of rate-limiting enzymes in glycolysis-gluconeogenesis that control the hepatic flux through the glucose/glucose-6-phosphate substrate cycle, GK and G6Pase, seems to play a critical role in glucose intolerance of carnivorous fish. *S. aurata* GK exhibits lower affinity for glucose than rat GK and delayed postprandial stimulation of GK mRNA levels in the liver [23]. On the other hand, the content of dietary carbohydrate fails to regulate the hepatic expression of G6Pase, which in turn is poorly repressed by insulin [24,25,26].

Despite *S. aurata* being a carnivorous fish, we previously showed that it tolerates partial substitution of dietary protein by carbohydrates through a metabolic adaptation that involves increased glucose oxidation, decreased gluconeogenesis and weaker transamination capacity in the liver [26,27,28,29,30]. Given that SREBP1 can promote a dual effect on the expression of lipogenic genes and genes encoding key enzymes in the regulation of glycolysis-gluconeogenesis, this transcription factor may be a unique target to facilitate conversion of dietary carbohydrates into lipids. In this study, with the aim to increase de novo lipogenesis from dietary carbohydrates in carnivorous fish, *S. aurata* juveniles were administered intraperitoneally chitosan-tripolyphosphate (TPP) nanoparticles complexed with a plasmid expressing the N-terminal transactivation domain of SREBP1a. The metabolic effect of SREBP1a overexpression was addressed in fish fed diets differing in macronutrient composition.

## 2. Materials and Methods

### 2.1. Animals

*S. aurata* juveniles obtained from Piscimar (Burriana, Castellón, Spain) with an average weight of 13.03 g ± 0.28 (mean ± SEM, *n* = 45) were maintained at 20 °C in 260-L aquaria supplied with running seawater as described [31]. Two groups of fish received isoenergetic diets differing in protein to carbohydrate ratio (HLL: high protein, low lipid, low carbohydrate; and LLH: low protein, low lipid, high carbohydrate). The composition of diets HLL and LLH is shown in Table 1. The diets were supplied at a ration of 40 g/kg body weight (BW) once a day (10:00) for 15 days. To study the effects of the expression of exogenous SREBP1a on *S. aurata* liver metabolism, fish fed diets HLL and LLH were intraperitoneally injected with chitosan-TPP nanoparticles complexed with a plasmid expressing the N-terminal active domain of hamster SREBP1a (pSG5-SREBP1a; [20]), or empty vector (pSG5; Agilent Technologies, Palo Alto, CA, USA) as a negative control, at a concentration of 10 μg of plasmid per gram BW. Seventy-two hours following the treatment and 24 h after the last meal, fish were sacrificed by cervical section, blood was collected and the liver was dissected out, frozen in liquid N_2_ and kept at −80 °C until use. To prevent stress, fish were anesthetised with tricaine methanesulfonate (MS-222; 78.4 mg/l) before handling. Experimental procedures involving fish were performed in accordance with the guidelines of the University of Barcelona’s Animal Welfare Committee (proceeding #461/16), in compliance with local legislation and EU Directive 2010/63/EU for animal experiments.

### 2.2. Chitosan-TPP-DNA Nanoparticles

Chitosan-TPP nanoparticles complexed with pSG5 or pSG5-SREBP1a were prepared following the ionic gelation method as previously described [32]. Low molecular weight chitosan (Sigma-Aldrich, St. Louis, MO, USA) was dissolved (2 mg/mL) in acetate buffer solution (pH 4.5) under magnetic stirring for 3 h and then filtered. Three-hundred microgram of pSG5 or pSG5-SREBP1a was dissolved in 1.2 mL of 0.84 mg/mL TPP (Sigma-Aldrich, St. Louis, MO, USA). TPP-DNA solutions were added dropwise to 3 mL of the chitosan-acetate buffer solution (1:0.4 chitosan:TPP ratio) under vigorous magnetic stirring. No DNA was used to obtain naked chitosan-TPP nanoparticles. Chitosan-TPP nanoparticles complexed in the presence and absence of DNA were sedimented by centrifugation at 19,000× *g* for 10 min at 15 °C, followed by washing twice with ultrapure water and resuspension in 2 mL of 2% *w/v* mannitol, which was used as a cryoprotector during lyophilisation. After a freeze-drying cycle at −47 °C, an additional drying step was carried out at 25 °C to remove residual water. Morphological characterisation of nanoparticles was performed by means of atomic force microscopy using peak force tapping mode (Multimode 8 AFM attached to a Nanoscope III Electronics, Bruker, USA). A Zetasizer Nano ZS fitted with a 633 nm laser (Malvern Instruments, Malvern, UK) was used to determine particle diameter by dynamic light scattering and *Z* potential of nanoparticles by laser Doppler electrophoresis. Chitosan-TPP-DNA nanoparticles were intraperitoneally administered to *S. aurata* after resuspension in 0.9 % NaCl.

### 2.3. Enzyme Activity Assays and Metabolites

Enzyme activity assays were performed in liver crude extracts obtained by homogenisation (1:5, *w/v*) of powdered frozen tissue in 50 mM Tris-HCl (pH 7.5), 4 mM, EDTA, 50 mM NaF, 0.5 mM phenylmethylsulfonyl fluoride, 1 mM dithiothreitol and 250 mM sucrose. Homogenisation was performed with a PTA-7 Polytron (Kinematica GmbH, Littau-Luzern, Switzerland) (position 3, 30 s). After centrifugation at 20,000 × *g* for 30 min at 4 °C, the supernatant was collected and used to perform enzyme activity assays. GK, 6-phosphofructo-1-kinase (PFK1), fructose-1,6-bisphosphatase (FBPase1) and total protein were assayed as described [23,27]. One unit of enzyme activity was defined as the amount of enzyme necessary for transforming 1 μmol of substrate per min, except for PFK1 activity, which expressed the amount of enzyme needed to oxidise 2 μmol of NADH per min. Enzyme activities were expressed per mg of soluble protein (specific activity). Glucose, triglycerides and cholesterol were measured with commercial kits (Linear Chemicals, Montgat, Barcelona, Spain). Spectrophotometric determinations were performed at 30 °C in a Cobas Mira S analyser (Hoffman-La Roche, Basel, Switzerland).

### 2.4. Reverse Transcriptase-Coupled Quantitative Real Time PCR (RT-qPCR)

Total RNA from the liver of *S. aurata* was isolated by means of RNeasy Tissue Mini Kit (QIAGEN, Sussex, UK) and reverse-transcribed with Moloney murine leukaemia virus RT (Life Technologies, Carlsbad, CA, USA) for 1 h at 37 °C in the presence of random hexamer primers. The mRNA levels of hamster SREBP1a and *S. aurata* GK, PFKFB1, acetyl-CoA carboxylase 1 (ACC1), acetyl-CoA carboxylase 2 (ACC2), fatty acid synthase (FASN), elongation of very long chain fatty acids protein 5 (ELOVL5), fatty acid desaturase 2 (FADS2), glucose-6-phosphate 1-dehydrogenase (G6PD) and 3-hydroxy-3-methylglutaryl-coenzyme A reductase (HMGCR) were determined in a Step One Plus Real-Time PCR System (Applied Biosystems, Foster City, CA, USA). The 20-μL mixture contained 0.4 μM of each primer (Table 2), 10 μL of SYBR Green (Applied Biosystems, Foster City, CA, USA), and 1.6 μL of diluted cDNA. The amplification cycle protocol was 95 °C for 10 min, followed by 40 cycles at 95 °C for 15 s and 62 °C for 1 min. Dissociation curves were applied after each experiment to confirm the amplification of single products. The size of amplicons was checked by agarose gel electrophoresis. Specificity of the reaction was assayed by sequencing amplicons at least once for each gene. Serial dilutions of control cDNA were used to generate standard curves in order to determine the efficiency of the amplification reaction for each gene. The mRNA levels for genes of interest in each sample were normalised using *S. aurata* ribosomal subunit 18s, β-actin and elongation factor 1 α (EF1α) as endogenous controls. The standard ΔΔC_T_ method was used to calculate variations in gene expression [33].

### 2.5. Western Blot

Fifty microgram of protein extract from liver samples was loaded per lane and submitted to electrophoresis in a 10 % sodium dodecyl sulfate–polyacrylamide gel electrophoresis gel. When electrophoresis was completed, the gel was equilibrated in transfer buffer (25 mM Tris-HCl, 192 mM glycine, 20 % methanol, pH 8.3), and electroelution proceeded at 4 °C onto NytranN nylon membranes (Whatman, Kent, UK) for 2 h at 60 V. A rabbit polyclonal antibody against rodent SREBP1 was used as the primary antibody (sc-8984, Santa Cruz Biotechnology, Dallas, TX, USA; 1:500). The Immun-Star Chemiluminescent Kit (Bio-Rad, Hercules, CA, USA) was used to immunodetect SREBP1 with an alkaline phosphatase-conjugated secondary antibody (Sigma-Aldrich, Saint Louis, MO, USA; 1:3000).

### 2.6. Obtention of Aqueous and Organic Fractions from the Liver

Organic and aqueous fractions were extracted from powdered frozen livers by the Folch method [34], with modifications. Briefly, 0.75 mL of methanol:water solution 2:1 (v/v) was added to 50 mg of liver sample and vortexed 1 min. Following the addition of 0.5 mL of chloroform and 0.25 mL of water (vortexing 1 min after every addition), and centrifugation at 2000× *g* for 20 min at 4 °C, upper and lower fractions were collected separately. The upper phase (methanol/water) contained water-soluble metabolites and was dried in a vacuum concentrator and stored at −20 °C until subsequent analysis by nuclear magnetic resonance (NMR). The lower phase (chloroform), containing lipids, was transferred to amber glass vials and dried under a nitrogen stream. After the addition of 2 mL hexane, samples were vortexed for 30 s and left to rest for 5 min to ensure fat dilution before adding 200 μL 2 N potassium hydroxide in methanol solution and centrifuging for 10 min at 2000× *g*. The upper phase was collected and kept at −80 °C until gas chromatography was performed.

### 2.7. NMR Analysis and Liver Metabolite Identification

The aqueous fractions were resuspended in 200 μL of 99.8 % ^2^H_2_O and 40 μL phosphate buffer (50 mM; pD 7.41; with 5 mM 3-(trimethylsilyl) propionic-2,2,3,3-d4 acid sodium salt and sodium azide in ^2^H_2_O). Samples were transferred into 3 mm NMR tubes. Proton (^1^H) NMR spectroscopy was conducted on a Varian VNMRS 600 MHz (Agilent, Santa Clara, CA, USA) spectrometer, equipped with a 3 mm ID-PFG broadband probe, at 298 K. Spectra were collected using a ^1^H-Presat pulse sequence (spectral width: 7200 Hz; relaxation delay: 4 s; saturation time: 3 s; acquisition time: 3 s). All spectra were processed with the ACD/NMR Processor Academic Edition from ACD\Labs 12.0 software (Advanced Chemistry Development, Inc.) applying: zero-filling to 65 k, line broadening of 0.2 Hz, phasing and baseline correction. The chemical shifts were referenced to TSP peak at 0 ppm. Metabolite identification and quantification was performed using ACD/NMR Processor Academic Edition from ACD\Labs 12.0 software (Advanced Chemistry Development, Inc.), assisted by the Chenomx NMR Suite Library version 10 (Chenomx Inc., Edmonton, Canada) and the Bayesil software [35].

### 2.8. Fatty Acid Methyl Ester (FAME) Analysis

Fatty acid composition was analysed as described [36], using a GC-2025 with autosampler (Shimadzu, Kyoto, Japan) equipped with flame ionization detector and BPX70, 30 m × 0.25 mm × 0.25 μm, capillary column (Trajan Scientific and Medical, Ringwood, Australia). The oven temperature was held 1 min at 60 °C and then raised to 260 °C at the rate of 6 °C/min. Injector (AOC-20i, Shimadzu, Kyoto, Japan) and detector temperatures were set at 260 °C and 280 °C, respectively. One microlitre of sample was injected with helium as carrier gas and split ratio 1:20. Fatty acids were identified by comparing FAME retention times with the Supelco 37 Component FAME Mix (Sigma-Aldrich, St. Louis, MO, USA).

### 2.9. Statistics

Analyses were performed with SPSS software Version 24 (IBM, Armonk, NY, USA). Data were submitted to two-way analysis of variance with diet (HLL vs. LLH) and treatment (control vs. SREBP1a-treated fish) as independent variables.

## 3. Results

### 3.1. Intraperitoneal Administration of Chitosan-TPP-pSG5-SREBP1a Increases mRNA and Immunodetectable Levels of SREBP1a in the Liver

**Figure 1:**
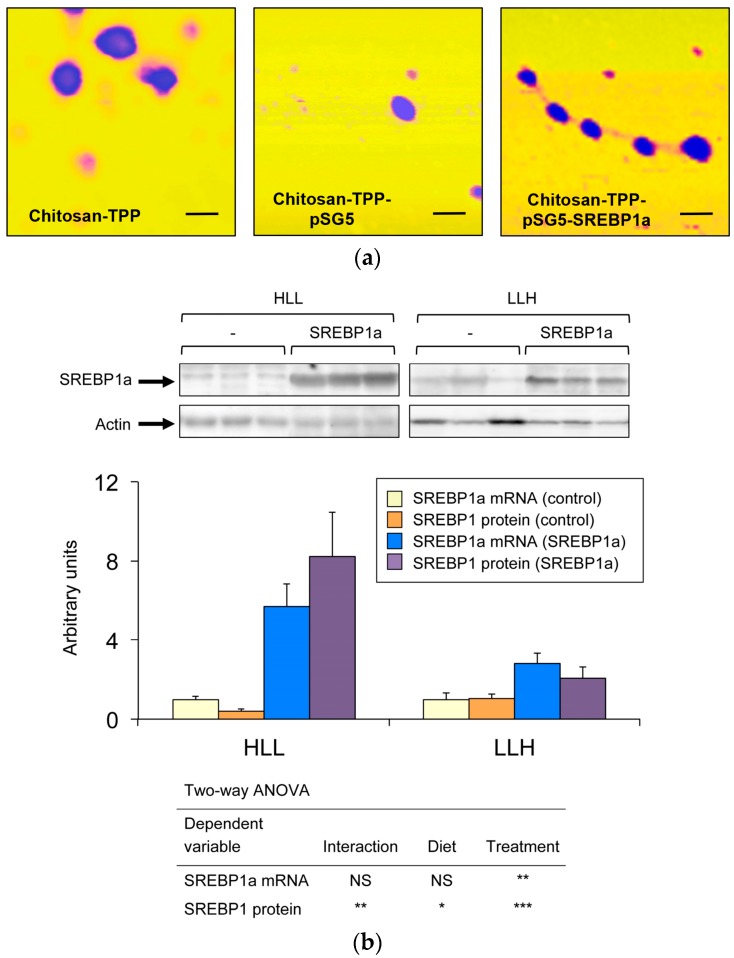
Effect of chitosan-TPP-pSG5-SREBP1a administration on SREBP1 mRNA and immunodetectable levels in the liver of *S. aurata*. (**a**) Representative images of chitosan-TPP, chitosan-TPP-pSG5 and chitosan-TPP-pSG5-SREBP1a nanoparticles obtained by atomic force microscopy. Black bars correspond to 200 nm. (**b**) The upper part of the panel shows a representative Western blot analysis of immunodetectable SREBP1 levels in liver extracts of of *S. aurata* fed diet HLL (high protein, low lipid, low carbohydrate) or LLH (low protein, low lipid, high carbohydrate) following 72 h of intraperitoneal administration with chitosan-TPP nanoparticles complexed with 10 μg/g BW of pSG5 (-) or pSG5-SREBP1a (SREBP1a). Two independent experiments were performed. The lower part of the panel shows the effect of chitosan-TPP-pSG5 (control) and chitosan-TPP-pSG5-SREBP1a (SREBP1a) nanoparticle administration on SREBP1 mRNA and protein levels in the liver of *S. aurata* fed diet HLL or LLH. Analysis of SREBP1 mRNA levels relative to the geometric mean of ribosomal subunit 18s, β-actin and EF1α were performed by RT-qPCR in liver samples of *S. aurata* at 72 h post-treatment. The values are expressed as mean ± standard error of the mean (SEM) (*n* = 6–7). Statistical significance for independent variables (diet and treatment) is indicated as follows: *p < 0.05; **p < 0.01; ***p < 0.001; *NS* not significant.

To study the metabolic effects resulting from overexpression of exogenous SREBP1a in the liver of *S. aurata*, ionic gelation was used to obtain chitosan-TPP nanoparticles complexed with an expression vector encoding the N-terminal nuclear fragment of hamster SREBP1a (pSG5-SREBP1a), which was shown to transactivate *S. aurata* GK and PFKFB1 genes in previous studies [20,21]. As a control, chitosan-TPP nanoparticles complexed with empty vector (pSG5) were also synthesised. Atomic force microscopy showed that chitosan-TPP nanoparticles presented rounded morphology, while dynamic light scattering indicated a mean diameter size of 230.9 nm ± 18.3 (mean ± standard error of the mean (SEM), *n* = 3). The mean *Z* potential was 31.2 mV ± 1.5 (mean ± SEM, *n* = 3). Incorporation of plasmid DNA to chitosan-TPP nanoparticles did not significantly affect morphology or diameter size, while decreased *Z* potential (Figure 1a). Mean diameter size and *Z* potential, were expressed as mean ± SEM, (*n* = 3), of chitosan-TPP-pSG5 were 233.6 nm ± 45.9 and 13.2 mV ± 0.4, respectively, while the values for chitosan-TPP-pSG5-SREBP1a were 272.3 nm ± 36.0 and 14.1 mV ± 2.3.

Twenty-four hours after the last meal, *S. aurata* fed HLL and LLH diets received an intraperitoneal injection of chitosan-TPP complexed with 10 μg/g BW of plasmid (pSG5-SREBP1a or empty vector, pSG5). To validate overexpression of SREBP1a in the liver of fish administered with chitosan-TPP-pSG5-SREBP1a, hepatic mRNA and immunodetectable levels of SREBP1a were determined 72 h post-treatment. As shown in Figure 1b, treatment with chitosan-TPP-pSG5-SREBP1a increased both mRNA abundance and the protein content of SREBP1a in the liver. A stronger effect was observed in fish fed HLL.

### 3.2. Effect of SREBP1a Overexpression on Serum and Aqueous Metabolites in the Liver

**Figure 2:**
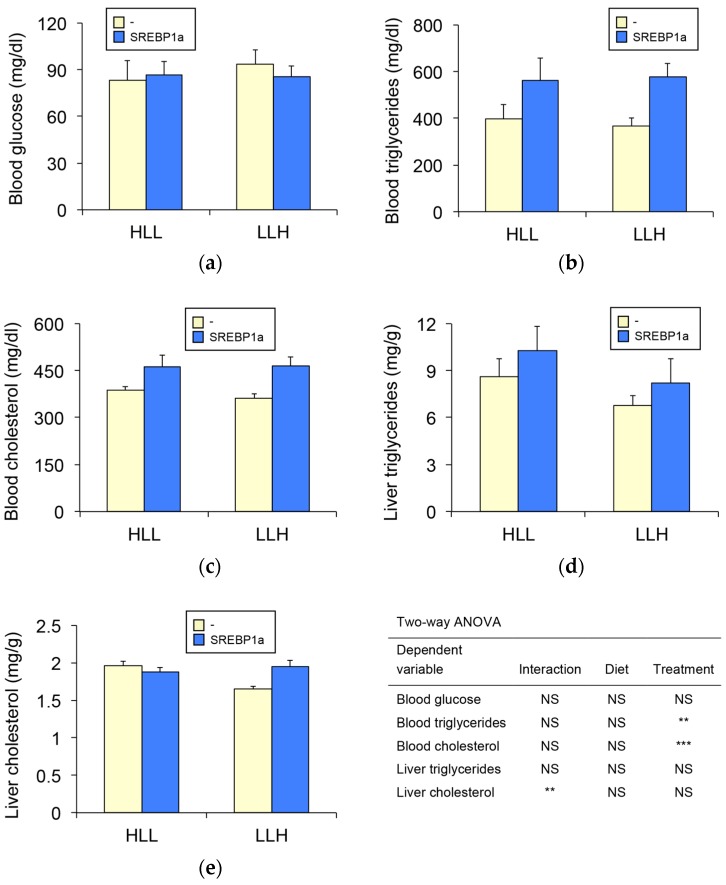
Effect of chitosan-TPP-pSG5-SREBP1a administration on serum and liver metabolite levels in *S. aurata*. Twenty-four h after the last meal, fish fed diet HLL or LLH were intraperitoneally administered with chitosan-TPP nanoparticles complexed with 10 μg/g BW of pSG5 (-) or pSG5-SREBP1a (SREBP1a). Seventy-two hours following the treatment, the liver and blood were collected. The levels of blood glucose (**a**), blood triglycerides (**b**), blood cholesterol (**c**), liver triglycerides (**d**) and liver cholesterol (**e**) are presented as mean ± SEM (*n* = 6–9). Statistical significance for independent variables (diet and treatment) is indicated as follows: **p < 0.01; ***p < 0.001; *NS* not significant.

Given that administration of chitosan-TPP-pSG5-SREBP1a significantly induced SREBP1a expression in the liver of *S. aurata*, we addressed the metabolic effects resulting from hepatic upregulation of SREBP1a. Concerning serum metabolites and regardless of the diet, SREBP1a overexpression significantly increased circulating triglycerides (1.2 to 1.3-fold) and cholesterol (1.4 to 1.6-fold), while did not affect blood glucose levels (Figure 2a–c). Although not significant, liver triglycerides also showed a trend to increase in SREBP1a-treated fish (Figure 2d), while only fish fed LLH presented higher cholesterol levels (Figure 2e).

^1^H-NMR studies allowed us to analyse the effect of SREBP1a overexpression in aqueous metabolites in the liver. Table 3 shows that 72 h post-treatment with chitosan-TPP-pSG5-SREBP1a nanoparticles significantly increased hepatic alanine (1.3 to 1.5-fold) and glycine (1.5 to 1.6-fold), while decreased formate (72 % to 80 % of the values observed in control animals, treated with empty vector). The SREBP1a-dependent effect on alanine levels was not affected by the diet. However, in addition to the effect of nanoparticle administration, the supply of the low protein, high carbohydrate diet (LLH) significantly increased glycine (2.2-fold) and formate (1.3-fold). Acetate levels significantly increased (1.2-fold) in the liver of *S. aurata* fed diet LLH, while they were not affected by SREBP1a overexpression.

### 3.3. Effect of SREBP1a Overexpression on Hepatic Glycolysis-Gluconeogenesis

**Figure 3:**
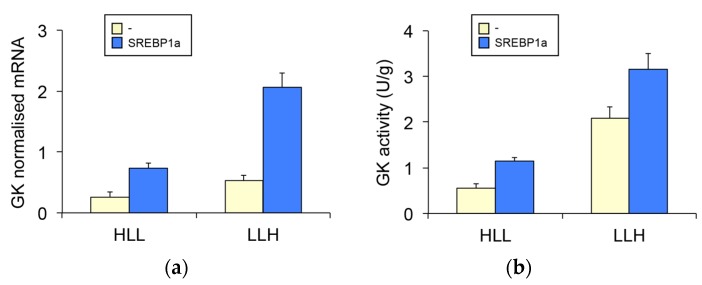

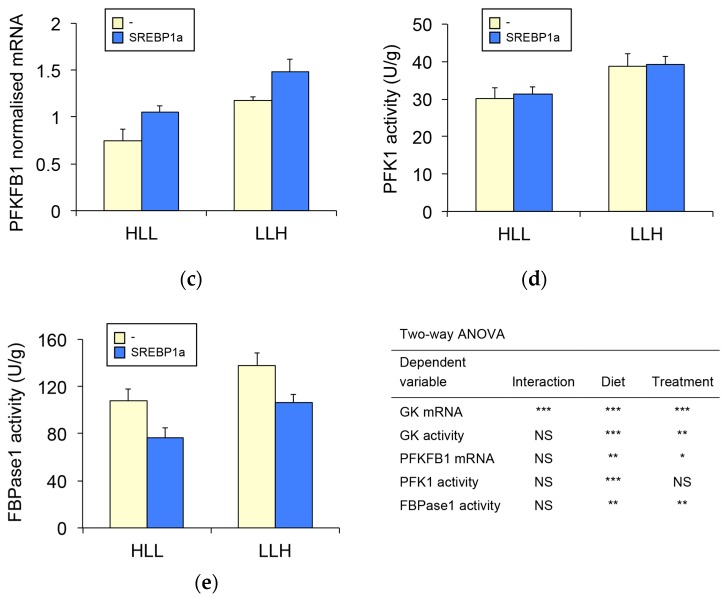
Effect of chitosan-TPP-pSG5-SREBP1a administration on the expression of key enzymes in glycolysis-gluconeogenesis in the liver of *S. aurata*. Twenty-four hours after the last meal, fish fed diet HLL or LLH were intraperitoneally administered with chitosan-TPP nanoparticles complexed with 10 μg/g BW of pSG5 (-) or pSG5-SREBP1a (SREBP1a). Seventy-two hours post-treatment, the liver was collected and RNA isolated. Hepatic mRNA levels and enzyme activity of GK (**a**, **b**), PFKFB1 (**c**), PFK1 (**d**) and FBPase1 (**e**) are presented as mean ± SEM (enzyme activity, *n* = 8; mRNA levels, *n* = 6). The mRNA levels for each gene were assayed by RT-qPCR and normalised with the geometric mean of ribosomal subunit 18s, β-actin and EF1α. Statistical significance for independent variables (diet and treatment) is indicated as follows: *p < 0.05; **p < 0.01; ***p < 0.001; *NS* not significant.

We previously reported that the N-terminal nuclear fragment of SREBP1a transactivates GK and PFKFB1 gene expression in *S. aurata* by binding to sterol regulatory element (SRE) boxes located in the proximal promoter of both genes [20,21]. Therefore, the fact that chitosan-TPP-pSG5-SREBP1a administration stimulated the expression of SREBP1a in the liver prompted us to analyse the effect of SREBP1a overexpression on hepatic mRNA levels of GK, a rate-limiting enzyme in glycolysis, and PFKFB1, a bifunctional enzyme that exerts an essential contribution to the control of glycolysis and gluconeogenesis through the synthesis and degradation of fructose-2,6-bisphosphate (fru-2,6-P_2_). In agreement with previous reports [26,28], a low protein, high carbohydrate diet (LLH) significantly increased GK and PFKFB1 mRNA levels in the liver of *S. aurata* by 2- and 1.6-fold, respectively (Figure 3a,c). In addition, SREBP1a overexpression upregulated the hepatic mRNA content of GK (2.8 to 3.9-fold) and PFKFB1 (1.3 to 1.4-fold). Consistently, GK activity significantly increased 1.5 to 2.1-fold as a result of SREBP1a overexpression (Figure 3b).

Given that SREBP1a-dependent upregulation of GK and PFKFB1 points to stimulation of the glycolytic flux in the liver, we also analysed the activity of PFK1 and FBPase1, which have a major role in the regulation of glycolysis-gluconeogenesis by catalysing the flux through the fructose-6-phosphate/fructose-1,6-bisphosphate substrate cycle. Meanwhile, diet LLH had a similar stimulatory effect on both enzymes, SREBP1a overexpression did not affected PFK1 (Figure 3d), but significantly decreased FBPase1 (71 % to 77 % of the values observed in control fish; Figure 3e).

### 3.4. SREBP1a Overexpression Enhances Hepatic Biosynthesis of Fatty Acids and Cholesterol

**Figure 4:**
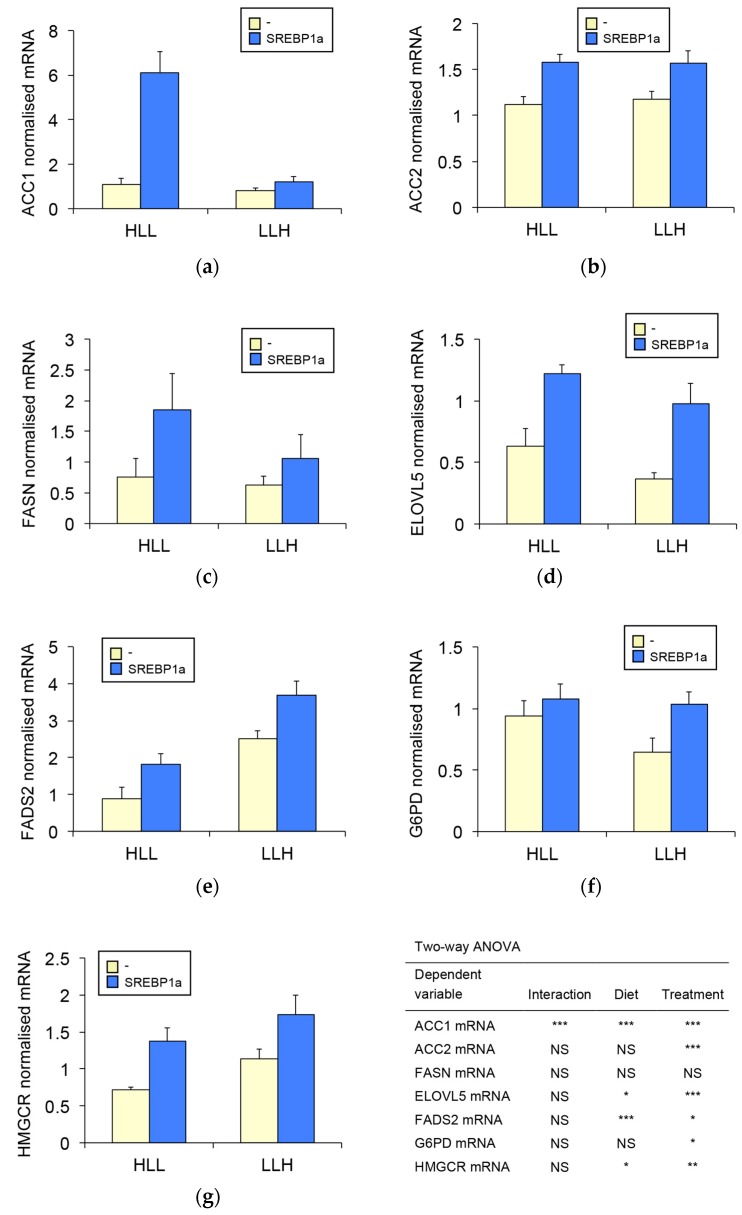
Effect of chitosan-TPP-pSG5-SREBP1a administration on the expression of key enzymes in the synthesis of fatty acid and cholesterol in the liver of *S. aurata*. Twenty-four hours after the last meal, fish fed diet HLL or LLH were intraperitoneally administered with chitosan-TPP nanoparticles complexed with 10 μg/g BW of pSG5 (-) or pSG5-SREBP1a (SREBP1a). Seventy-two hours following the treatment, the liver was collected and RNA isolated. Hepatic mRNA levels of ACC1 (**a**), ACC2 (**b**), FASN (**c**), ELOVL5 (**d**), FADS2 (**e**), G6PD (**f**) and HMGCR (**g**) are presented as mean ± SEM (*n* = 6). Expression levels for each gene were assayed by RT-qPCR and normalised with the geometric mean of ribosomal subunit 18s, β-actin and EF1α. Statistical significance for independent variables (diet and treatment) are indicated as follows: *p < 0.05; **p < 0.01; ***p < 0.001; *NS* not significant.

Due to the well-known stimulatory effect of SREBP1a on the expression of genes involved in biosynthesis of fatty acids and cholesterol [1], we studied the effect of chitosan-TPP-pSG5-SREBP1a administration on gene expression levels of key enzymes involved in lipid biosynthesis in the liver of *S. aurata*. SREBP1a overexpression significantly upregulated mRNA levels of most genes analysed: rate-limiting enzymes in fatty acid synthesis (ACC1 and ACC2, Figure 4a–c), genes involved in fatty acid elongation (ELOVL5, Figure 4d) and desaturation (FADS2, Figure 4e) as well as key enzymes for the provision of NADPH (G6PD, Figure 4f) and cholesterol synthesis (HMGCR, Figure 4g). The highest stimulatory effect of SREBP1a was found for the expression levels of ACC1, which increased 5.6-fold in *S. aurata* fed diet HLL.

Table 4 shows changes in the fatty acid profile in the liver of *S. aurata* treated with chitosan-TPP-pSG5-SREBP1a. All fatty acids that were significantly affected by the treatment (seven out of 29), increased their content in the liver of fish submitted to SREBP1a overexpression. Fatty acids affected by SREBP1a included pentadecanoic acid (15:0-n), heptadecanoic acid (17:0-n), myristoleic acid (14:1n-5), *cis*-10-pentadecenoic acid (15:1n-5) and long-chain polyunsaturated fatty acids (LC-PUFA), such as *cis*-11,14-eicosadienoic acid (20:2n-6), *cis*-11,14,17-eicosatrienoic acid (20:3n-3) and *cis*-13,16-docosadienoic acid (22:2n-6). Most remarkable SREBP1a-dependent increases were found for pentadecanoic acid (1.1 to 2.9-fold), *cis*-11,14-eicosadienoic acid (1.3 to 1.4-fold) and *cis*-11,14,17-eicosatrienoic acid (1.4-fold) as well as for myristoleic acid, *cis*-10-pentadecenoic acid and *cis*-13,16-docosadienoic acid, which presented barely detectable levels in non-treated *S. aurata* fed diet LLH.

## 4. Discussion

Chitosan is a cationic polymer derived from chitin that is composed of glucosamine and N-acetylglucosamine units linked by β-1,4-glycosidic bonds. Properties such as mucoadhesion, low toxicity, biodegradability, biocompatibility and protection of nucleic acids against nuclease degradation have made chitosan a valuable gene-carrier system to deliver nucleic acids in vivo [37,38]. In recent studies, we used the ionic gelation technique to obtain chitosan-TPP nanoparticles complexed with plasmids expressing short hairpin RNAs specifically designed to knockdown the expression of target genes in the liver of *S. aurata*. By using this methodology we were able to study the metabolic effect that results from silencing the expression of cytosolic alanine aminotransferase and glutamate dehydrogenase [32,39]. Herein, we obtained chitosan-TPP nanoparticles complexed with a plasmid that expresses the N-terminal activation domain of SREBP1a (pSG5-SREBP1a). The metabolic effect of SREBP1a overexpression was addressed in fish fed diets differing in macronutrient composition.

As a cationic polymer, it is considered that chitosan can absorb fatty acids and lower circulating triglycerides. In the present study, no significant differences were found for blood and liver triglycerides between non-treated *S. aurata* and fish treated with chitosan-TPP-pSG5 nanoparticles, which supports the notion that under our experimental conditions chitosan did not affect the hepatic content of fatty acids (Supplementary Table S1). The lack of effect due to chitosan may be attributed to the animal model and experimental design of the present study, where fish received a single dose of chitosan by intraperitoneal injection. This contrasts with reports that showed a chitosan-dependent lowering effect on circulating triglycerides, which have been essentially performed in mammals, mostly in rats submitted to an oral dose or fed with chitosan-containing diets for a period of time [40,41].

Intraperitoneal administration of chitosan-TPP-pSG5-SREBP1a significantly increased mRNA and immunodetectable levels of SREBP1a in the liver of *S. aurata*. The presence of SREBP1-stabilising factors in the liver of fish fed high protein, low carbohydrate diets may explain higher amounts of immunodetectable SREBP1 in fish fed HLL. Likewise, we previously showed that partial substitution of dietary protein by carbohydrates decreases body lipids and growth performance in *S. aurata* [29]. Consistent with previous findings that reported transactivation of *S. aurata* GK and PFKFB1 by SREBP1a [20,21], overexpression of SREBP1a upregulated the expression of both genes even in the group of fish fed the high carbohydrate/low protein diet, were in agreement with previous reports [26,28], dietary carbohydrates enhanced basal expression of glycolytic genes in the liver of *S. aurata*.

Enhancement of GK activity may be responsible for increasing hepatic glycogen, a trend that was found in the liver of fish treated with chitosan-TPP-pSG5-SREBP1a nanoparticles. Increased GK activity is needed to convert glucose into glucose-6-phosphate but may not be sufficient to increase the glycolytic flux. However, our findings support the notion that in addition to allocate part of the glucose-6-phosphate pool resulting from increased GK activity to synthesise glycogen, SREBP1a-dependent upregulation of PFKFB1 would be a key mechanism to favour glucose oxidation via glycolysis in the liver of fish that overexpressed SREBP1a. Consistently, glucose and insulin administration increases SREBP1 protein content and association with PFKFB1 promoter in the liver of *S. aurata*, leading to PFKFB1 upregulation [20]. PFKFB1 is a bifunctional enzyme that catalyses the synthesis and degradation of fru-2,6-P_2_, which, in turn, is a major regulator of glycolysis–gluconeogenesis through allosteric activation of PFK1 and inhibition of FBPase1 [42,43]. Therefore, by controlling the activity of PFK1 and FBPase1, the cytosolic levels of fru-2,6-P_2_ are essential to determine the flux through the fructose-6-phosphate/fructose-1,6-bisphosphate substrate cycle.

In *S. aurata*, we previously reported that high carbohydrate diets upregulate the hepatic mRNA levels of PFKFB1 and the kinase activity of the bifunctional enzyme, leading to a huge increase of fru-2,6-P_2_ [28]. In the present study, the effect of SREBP1a overexpression was also assessed in the activity of the two enzymes that regulate the fructose-6-phosphate/fructose-1,6-bisphosphate substrate cycle: PFK1 and FBPase1. Consistent with increased expression of PFKFB1 and conceivably the fru-2,6-P_2_ content, FBPase1 activity was significantly reduced in the liver of treated fish, which suggests inhibition of the gluconeogenic pathway. Surprisingly, PFK1 activity was not affected by overexpression of SREBP1a. Similarly as in mammals, fru-2,6-P_2_ behaves as allosteric activator of *S. aurata* PFK-1 in vitro [44]. However, the concomitant occurrence of other molecules that can act as allosteric inhibitors of PFK1, such as ATP and citrate, may counteract the stimulatory action of fru-2,6-P_2_ in the liver of *S. aurata* overexpressing SREBP1a. In support of this hypothesis, it was shown that the liver isoform of PFK1 is highly susceptible to inhibition by ATP in *S. aurata* [44]. Nevertheless, considering the effect of SREBP1a overexpression on the PFK1/FBPase1 activity ratio, our findings suggest that by decreasing the gluconeogenic activity, SREBP1a favoured the glycolytic flux through the fructose-6-phosphate/fructose-1,6-bisphosphate substrate cycle. Increased levels of alanine and glycine in the liver of *S. aurata* support the enhancement of hepatic glycolysis from dietary carbohydrates as a result of SREBP1a overexpression. An increased glycolytic rate would favour further conversion of pyruvate into alanine and the use of glycolytic intermediates for biosynthetic purposes, such as 3-phosphoglycerate for subsequent biosynthesis of serine and glycine.

The results of this study suggest that SREBP1a-dependent increased glucose oxidation via glycolysis can assure a steady provision of carbons for de novo lipogenesis in the liver of *S. aurata*. Consistently, treatment with chitosan-TPP-pSG5-SREBP1a nanoparticles significantly upregulated the expression of key genes involved in biosynthesis of fatty acids and cholesterol, leading to increased circulating levels of triglycerides and cholesterol. In agreement with the role of SREBP1 in the transcription of lipogenic genes in mammals and fish [6,45,46,47,48,49], overexpression of SREBP1a in the liver of treated fish upregulated the expression of genes involved in synthesis (ACC1 and ACC2), elongation (ELOVL5) and desaturation (FADS2) of fatty acids, NADPH production (G6PD) and cholesterol biosynthesis (HMGCR).

By providing malonyl-CoA for the biosynthesis of fatty acids, ACC1 catalyses the rate-limiting step in fatty acid synthesis [50,51]. In the present study, the effect of SREBP1a overexpression on ACC1 was potentiated in fish fed the higher protein/lower carbohydrate diet. Albeit not significant, the same trend was observed for another important enzyme in fatty acid synthesis, FASN, which generates palmitate (16:0) from acetyl-CoA, malonyl-CoA and NADPH. Altogether, our findings suggest that dietary protein may either increase SREBP1a stability or have a synergistic effect with SREBP1a in the transcriptional activation of ACC1 and even FASN. In this regard, it is well known that in mammals insulin activates SREBP1 cleavage and production of the nuclear mature form of the protein through signalling pathways involving Akt and mTORC1 [7,52]. A similar behaviour may occur in carnivorous fish, where amino acids are considered the most potent insulin secretagogues [22]. Furthermore, consistent with our findings, it was proposed that dietary protein might enhance lipogenesis via mTOR signalling in rainbow trout [53,54,55].

SREBP1a-dependent activation of lipogenic genes caused significant changes in the hepatic fatty acid profile of treated fish. Upregulation of ACC1 and FASN combined with increased expression of genes involved in fatty acid elongation and desaturation significantly increased the relative concentration of a wide array of fatty acids, which is consistent with the fact that the activity of FADS2 and ELOVL5 can desaturate and condense, respectively, a broad range of fatty acids [56,57,58]. This seems to be also the case in fish species, where FADS2 enzymes exhibit predominantly Δ6 desaturase activity but display more varied substrate specificity than in mammals, while piscine ELOVL5 shows more versatility than its mammalian orthologues [59]. Affected fatty acids included saturated, monounsaturated and PUFAs. However, SREBP1a overexpression did not change the total content of saturated and unsaturated fatty acids as well as the n-6/n-3 ratio.

Even though SREBPs are conserved from yeast to humans, it remains uncertain to what extent the results of the present study can be generalised to mammals and other vertebrates. Physiological and metabolic features of fish, such as dependence on dietary amino acids to produce energy and adaptation to long-term food deprivation, and the fact that species-specific differences in SREBP targets and control pathways have been reported [7], make comparison between carnivorous fish and other vertebrates difficult.

## 5. Conclusions

In this study we show that treatment with chitosan-TPP-pSG5-SREBP1a nanoparticles led to SREBP1a overexpression in the liver, which in turn promoted a multigenic action that increased the glycolytic flux by stimulating GK and PFKFB1 expression as well as lipogenesis through upregulation of genes involved in biosynthesis of fatty acids and cholesterol. In addition to report for the first time the in vivo effects of exogenous SREBP1a in a glucose-intolerant model, our findings support that SREBP1a overexpression in the liver can trigger a protein sparing effect through conversion of dietary carbohydrates into lipids in *S. aurata*.

## Figures and Tables

**Table 1:** Composition of the diets supplied in this study to *S. aurata*.

	HLL	LLH
Formulation (%)		
Fish meal ^1^	81.6	54.3
Fish oil ^2^	0.8	6.0
Starch ^3^	15.0	37.1
Vitamin mixture ^4^	0.2	0.2
Mineral mixture ^5^	0.9	0.9
Carrageenan ^6^	1.5	1.5
Proximate composition (%)		
Protein	58.0	38.6
Carbohydrates ^7^	15.0	37.1
Fat	9.9	12.1
Ash	15.4	10.5
Gross energy (kJ/g) ^8^	20.1	20.0

^1^ Corpesca S.A. Super-Prime fish meal (Santiago de Chile, Chile). ^2^ Fish oil from A.F.A.M.S.A. (Vigo, Spain). ^3^ Pregelatinised corn starch from Brenntag Química S.A. (St. Andreu de la Barca, Barcelona, Spain). ^4^ Vitamin mixture provided (mg/kg): choline chloride, 1200; myo-inositol, 400; ascorbic acid, 200; nicotinic acid, 70; all-rac-tocopherol acetate, 60; calcium pantothenate, 30; riboflavin, 15; piridoxin, 10; folic acid, 10; menadione, 10; thiamin-HCl, 8; all-trans-retinol, 2; biotin, 0,7 cholecalciferol, 0.05; cyanocobalamin, 0.05. ^5^ Mineral mixture provided (mg/kg): CaHPO_4_.2H_2_O, 7340; MgO, 800; KCl, 750; FeSO_4_.7H_2_O, 60; ZnO, 30; MnO_2_, 15; CuSO_4_.5H_2_O, 1.7; CoCl_2_.6H_2_O, 1.5; KI, 1.5; Na_2_SeO_3_, 0.3. ^6^ Iota carrageenan (Sigma-Aldrich). ^7^ Carbohydrates were calculated by difference. ^8^ Calculated from the gross composition (protein 24 kJ/g, lipids 39 kJ/g, carbohydrates 17 kJ/g).

**Table 2:** Primer sequences used for reverse transcriptase-coupled quantitative real time PCR (RT-qPCR) in the present study.

Gene	Forward Sequences (5’ to 3’)	Reverse Sequences (5’ to 3’)	GenBank Accession
SREBP1a	CCTCCTGCCTCCGAGTTTCC	GAAGGAAGGCTAGAATACCCC	U09103
GK	TGTGTCAGCTCTCAACTCGACC	AGGATCTGCTCTACCATGTGGAT	AF169368
PFKFB1	TCGTGATGGTGGGACTGCCG	CTCGGCGTTGTCGGCTCTGAAG	U84724
ACC1	CCCAACTTCTTCTACCACAG	GAACTGGAACTCTACTACAC	JX073712
ACC2	TGACATGAGTCCTGTGCTGG	GCCTCAGTTCGTATGATGGT	JX073714
FASN	TGGGTCAGGGGGAGTTGG	TTGTGTGGAGAAGAACGTGCTGT	JQ277708
ELOVL5	GGGATGGCTACTGCTCGACA	CAGGAGAGTGAGGCCCAGAT	AY660879
FADS2	CACTATGCTGGAGAGGATGCC	TATTTCGGTCCTGGCTGGGC	AY055749
G6PD	TGATGATCCAACAGTTCCTA	GCTCGTTCCTGACACACTGA	JX073711
HMGCR	ACTGATGGCTGCTCTGGCTG	GGGACTGAGGGATGACGCAC	MN047456
18s	TTACGCCCATGTTGTCCTGAG	AGGATTCTGCATGATGGTCACC	AM490061
β-actin	CTGGCATCACACCTTCTACAACGAG	GCGGGGGTGTTGAAGGTCTC	X89920
EF1α	CCCGCCTCTGTTGCCTTCG	CAGCAGTGTGGTTCCGTTAGC	AF184170

**Table 3:** Effect of SREBP1a overexpression on aqueous metabolites in the liver of *S. aurata*.

	HLL		LLH		Two-way ANOVA		
Metabolite (mM)	-	SREBP1a	-	SREBP1a	Interaction	Diet	Treatment
Lactate + Thr	0.96 ± 0.16	1.13 ± 0.19	0.78 ± 0.16	1.20 ± 0.35	NS	NS	NS
Alanine	3.21 ± 0.67	4.12 ± 0.72	2.87 ± 0.29	4.35 ± 0.31	NS	NS	*
Glucose	3.22 ± 0.44	4.44 ± 0.98	1.87 ± 0.31	3.27 ± 1.12	NS	NS	NS
Acetate	0.22 ± 0.02	0.21 ± 0.03	0.27 ± 0.02	0.28 ± 0.03	NS	*	NS
Isoleucine	0.04 ± 0.01	0.03 ± 0.01	0.03 ± 0.00	0.04 ± 0.01	NS	NS	NS
Valine	0.08 ± 0.01	0.08 ± 0.02	0.07 ± 0.00	0.07 ± 0.01	NS	NS	NS
Creatinine	0.09 ± 0.02	0.09 ± 0.01	0.29 ± 0.05	0.23 ± 0.05	***	NS	NS
Choline compounds	0.44 ± 0.03	0.43 ± 0.08	0.36 ± 0.03	0.39 ± 0.03	NS	NS	NS
Succinate	0.37 ± 0.05	0.45 ± 0.09	0.24 ± 0.04	0.38 ± 0.09	NS	NS	NS
Sarcosine	0.07 ± 0.01	0.07 ± 0.01	0.05 ± 0.01	0.05 ± 0.01	NS	NS	NS
Taurine	6.46 ± 0.45	5.51 ± 0.75	5.77 ± 0.26	4.66 ± 0.40	NS	NS	NS
Glycine	0.86 ± 0.09	1.34 ± 0.20	1.90 ± 0.25	2.75 ± 0.38	NS	***	*
Formate	1.31 ± 0.11	0.94 ± 0.14	1.66 ± 0.18	1.32 ± 0.10	NS	*	*
Glycogen	1.28 ± 0.30	1.75 ± 0.16	1.84 ± 0.61	2.05 ± 0.63	NS	NS	NS
Lac/Ala	0.31 ± 0.03	0.28 ± 0.03	0.27 ± 0.03	0.26 ± 0.06	NS	NS	NS

Aqueous metabolites were assayed by ^1^H-NMR in liver extracts of *S. aurata* fed diets HLL or LLH 72 h following administration of chitosan-TPP nanoparticles complexed with 10 μg/g BW of pSG5 (-) or pSG5-SREBP1a (SREBP1a). The values are expressed as mean ± SEM (*n* = 5). Statistical significance for independent variables (diet and treatment) related to control fish (empty vector, pSG5) is indicated as follows: *p < 0.05; ***p < 0.001; *NS*, not significant.

**Table 4:** Effect of SREBP1a overexpression on fatty acid profile in the liver of *S. aurata*.

	HLL		LLH		Two-way ANOVA		
Fatty Acid	-	SREBP1a	-	SREBP1a	Interaction	Diet	Treatment
14:0	3.59 ± 0.31	3.10 ± 0.15	2.85 ± 0.12	2.76 ± 0.44	NS	NS	NS
15:0	0.28 ± 0.05	0.30 ± 0.02	0.11 ± 0.00	0.32 ± 0.01	*	*	**
16:0	22.26 ± 0.76	21.32 ± 0.54	22.09 ± 1.11	23.73 ± 0.96	NS	NS	NS
17:0	0.32 ± 0.03	0.41 ± 0.01	0.33 ± 0.01	0.35 ± 0.01	NS	NS	*
18:0	7.14 ± 1.05	7.65 ± 0.37	8.69 ± 0.30	6.93 ± 0.37	NS	NS	NS
20:0	0.10 ± 0.02	0.12 ± 0.00	0.11 ± 0.01	0.10 ± 0.01	NS	NS	NS
21:0	0.89 ± 0.14	1.21 ± 0.14	1.21 ± 0.47	1.27 ± 0.14	NS	NS	NS
22:0	0.15 ± 0.02	0.16 ± 0.02	0.13 ± 0.02	0.16 ± 0.00	NS	NS	NS
23:0	0.01 ± 0.01	0.03 ± 0.01	0.00 ± 0.00	0.01 ± 0.01	NS	*	NS
24:0	0.13 ± 0.01	0.15 ± 0.02	0.18 ± 0.06	0.18 ± 0.02	NS	NS	NS
14:1n-5	0.09 ± 0.02	0.11 ± 0.01	0.00 ± 0.00	0.11 ± 0.04	NS	NS	*
15:1n-5	0.07 ± 0.02	0.09 ± 0.01	0.00 ± 0.00	0.08 ± 0.02	NS	*	*
16:1n-7	4.42 ± 0.26	4.53 ± 0.35	4.25 ± 0.14	3.96 ± 0.20	NS	NS	NS
17:1n-7	0.28 ± 0.02	0.32 ± 0.01	0.29 ± 0.05	0.33 ± 0.01	NS	NS	NS
18:1n-9c	26.48 ± 0.95	27.15 ± 1.83	26.10 ± 0.40	23.69 ± 1.53	NS	NS	NS
18:1n-9t	0.11 ± 0.01	0.12 ± 0.01	0.10 ± 0.02	0.10 ± 0.01	NS	NS	NS
20:1n-9	1.29 ± 0.14	1.40 ± 0.01	1.70 ± 0.03	1.40 ± 0.08	NS	NS	NS
22:1n-9	0.74 ± 0.08	0.80 ± 0.13	0.96 ± 0.07	1.05 ± 0.08	NS	*	NS
24:1n-9	0.30 ± 0.04	0.39 ± 0.04	0.31 ± 0.06	0.24 ± 0.02	NS	NS	NS
18:2n-6c	2.12 ± 0.38	2.37 ± 0.59	2.09 ± 0.05	2.43 ± 0.07	NS	NS	NS
18:2n-6t	0.01 ± 0.01	0.02 ± 0.00	0.02 ± 0.02	0.03 ± 0.01	NS	NS	NS
18:3n-3	0.11 ± 0.03	0.10 ± 0.03	0.11 ± 0.03	0.10 ± 0.01	NS	NS	NS
18:3n-6	0.36 ± 0.05	0.42 ± 0.09	0.32 ± 0.04	0.39 ± 0.03	NS	NS	NS
20:2n-6	0.13 ± 0.02	0.17 ± 0.01	0.14 ± 0.02	0.19 ± 0.01	NS	NS	*
20:3n-3	0.17 ± 0.02	0.24 ± 0.01	0.25 ± 0.04	0.36 ± 0.04	NS	*	*
20:4n-6	1.02 ± 0.07	0.96 ± 0.10	0.94 ± 0.02	1.38 ± 0.19	NS	NS	NS
20:5n-3	4.77 ± 0.14	4.27 ± 0.49	3.64 ± 0.22	4.98 ± 0.53	NS	NS	NS
22:2n-6	0.01 ± 0.00	0.03 ± 0.00	0.00 ± 0.00	0.02 ± 0.01	NS	NS	*
22:6n-3	14.46 ± 1.18	14.87 ± 1.84	12.46 ± 0.68	15.31 ± 1.16	NS	NS	NS
Saturated	35.48 ± 2.86	34.45 ± 0.85	35.76 ± 0.93	35.81 ± 0.74	NS	NS	NS
Monounsaturated	34.50 ± 0.52	34.91 ± 2.12	33.71 ± 0.54	30.95 ± 1.79	NS	NS	NS
PUFAs	23.01 ± 1.38	23.47 ± 3.11	19.99 ± 0.80	25.20 ± 1.83	NS	NS	NS
n-9	29.90 ± 0.52	29.86 ± 2.00	29.17 ± 0.46	26.47 ± 1.61	NS	NS	NS
n-6	3.49 ± 0.40	3.97 ± 0.79	3.53 ± 0.04	4.45 ± 0.18	NS	NS	NS
n-3	19.52 ± 1.23	19.49 ± 2.34	16.46 ± 0.82	20.75 ± 1.68	NS	NS	NS
n-6/n-3	0.18 ± 0.02	0.20 ± 0.02	0.22 ± 0.01	0.22 ± 0.01	NS	NS	NS

Fatty acid composition was assayed in liver extracts of *S. aurata* fed diets HLL or LLH 72 h following administration of chitosan-TPP nanoparticles complexed with 10 μg/g BW of pSG5 (-) or pSG5-SREBP1a (SREBP1a). Two replicate analyses per sample were performed and the results are expressed in % area as mean values ± SEM of five samples per condition. Statistical significance for independent variables (diet and treatment) related to control fish (empty vector, pSG5) is indicated as follows: *p < 0.05; **p < 0.01; *NS*, not significant.

## Supplementary Materials

The following are available online at https://www.mdpi.com/2218-273X/9/8/297/s1, Table S1: Triglyceride levels in serum and liver of non-treated *S. aurata* and *S. aurata* treated with chitosan-TPP-pSG5 and chitosan-TPP-pSG5-SREBP1a.
