# Supplementary material for: The Administration of Chitosan-Tripolyphosphate-DNA Nanoparticles to Express Exogenous SREBP1a Enhances Conversion of Dietary Carbohydrates into Lipids in the Liver of Sparus aurata
Source: Biomolecules. 2019 Jul 24;9(8):297. doi: 10.3390/biom9080297 (PMC6724022; doi:10.3390/biom9080297)
Supplement: Supplementary file 1 [file biomolecules-09-00297-s001.pdf]

Article

The administration of chitosan-tripolyphosphate-DNA nanoparticles to express exogenous SREBP1a enhances conversion of dietary carbohydrates into lipids in the liver of *Sparus aurata*

Jonás I. Silva-Marrero [1], Juliana Villasante [1,2], Ania Rashidpour [1], Mariana Palma [3], Anna Fàbregas [4], María Pilar Almajano [2], Ivan Viegas [3,5], John G. Jones [5], Montserrat Miñarro [4], Josep R. Ticó [4], Isabel V. Baanante [1] and Isidoro Metón [1,*]

[1] Secció de Bioquímica i Biologia Molecular, Departament de Bioquímica i Fisiologia, Facultat de Farmàcia i Ciències de l'Alimentació, Universitat de Barcelona, Joan XXIII 27-31, 08028 Barcelona, Spain
[2] Departament d'Enginyeria Química, Universitat Politècnica de Catalunya, Diagonal 647, 08028 Barcelona, Spain
[3] Center for Functional Ecology (CFE), Department Life Sciences, University of Coimbra, Calçada Martins de Freitas 3000-456 Coimbra, Portugal
[4] Departament de Farmàcia i Tecnologia Farmacèutica, i Fisicoquímica, Facultat de Farmàcia i Ciències de l'Alimentació, Universitat de Barcelona, Joan XXIII 27-31, 08028 Barcelona, Spain
[5] Center for Neuroscience and Cell Biology (CNC), University of Coimbra, Largo Marquês de Pombal, 3004-517 Coimbra, Portugal
* Correspondence: imeton@ub.edu; Tel.: +34-93-4024521 (I.M.)

Supplementary materials

Table S1. Triglyceride levels in serum and liver of non-treated *S. aurata* and *S. aurata* treated with chitosan-TPP-pSG5 and chitosan-TPP-pSG5-SREBP1a.

	**HLL**		**LLH**	
	Serum (mg/dl)	Liver (mg/g)	Serum (mg/dl)	Liver (mg/g)
Non-treated	262.2 ± 88.0 [a]	7.86 ± 1.14	260.4 ± 78.7 [a]	6.58 ± 1.40
Chitosan-TPP-pSG5	399.6 ± 62.3 [a]	8.61 ± 1.40	368.4 ± 66.6 [a]	6.77 ± 1.40
Chitosan-TPP-pSG5-SREBP1a	561.4 ± 71.9 [b]	10.28 ± 1.14	577.3 ± 71.9 [b]	8.19 ± 1.14

Triglycerides were determined in serum and liver extracts of *S. aurata* fed diets HLL or LLH 72 h following no treatment or administration of chitosan-TPP nanoparticles complexed with 10 µg/g BW of pSG5 or pSG5-SREBP1a (SREBP1a). The values are expressed as mean ± SEM (n=4-8). Data were submitted to two-way ANOVA with diet (HLL and LLH) and treatment (non-treated, chitosan-TPP-pSG5 and chitosan-TPP-pSG5-SREBP1a) as independent variables. Different superscript letters indicate significant differences among treatments for a given diet according to the Student-Newman-Keuls post hoc test ($P < 0.05$). No statistical significance was found for diet and the interaction between diet and treatment.
